# Unmasking a fungal fire

**DOI:** 10.1371/journal.ppat.1011355

**Published:** 2023-05-18

**Authors:** Ivy M. Dambuza, Adilia Warris, Fabián Salazar

**Affiliations:** MRC Centre for Medical Mycology, University of Exeter, Exeter, United Kingdom; University of Maryland, Baltimore, UNITED STATES

## Abstract

Immune checkpoint inhibitor (ICI) therapy represents a breakthrough cancer treatment by stimulating dysfunctional T cells in the tumour environment to kill cancer cells. Beyond effects on anticancer immunity, ICI therapy may be associated with increased susceptibility to or more rapid resolution of chronic infections, particularly those caused by human fungal pathogens. In this concise review, we summarise recent observations and findings that implicate immune checkpoint blockade in fungal infection outcomes.

## 1. How does ICI therapy work?

**Fig 1:**
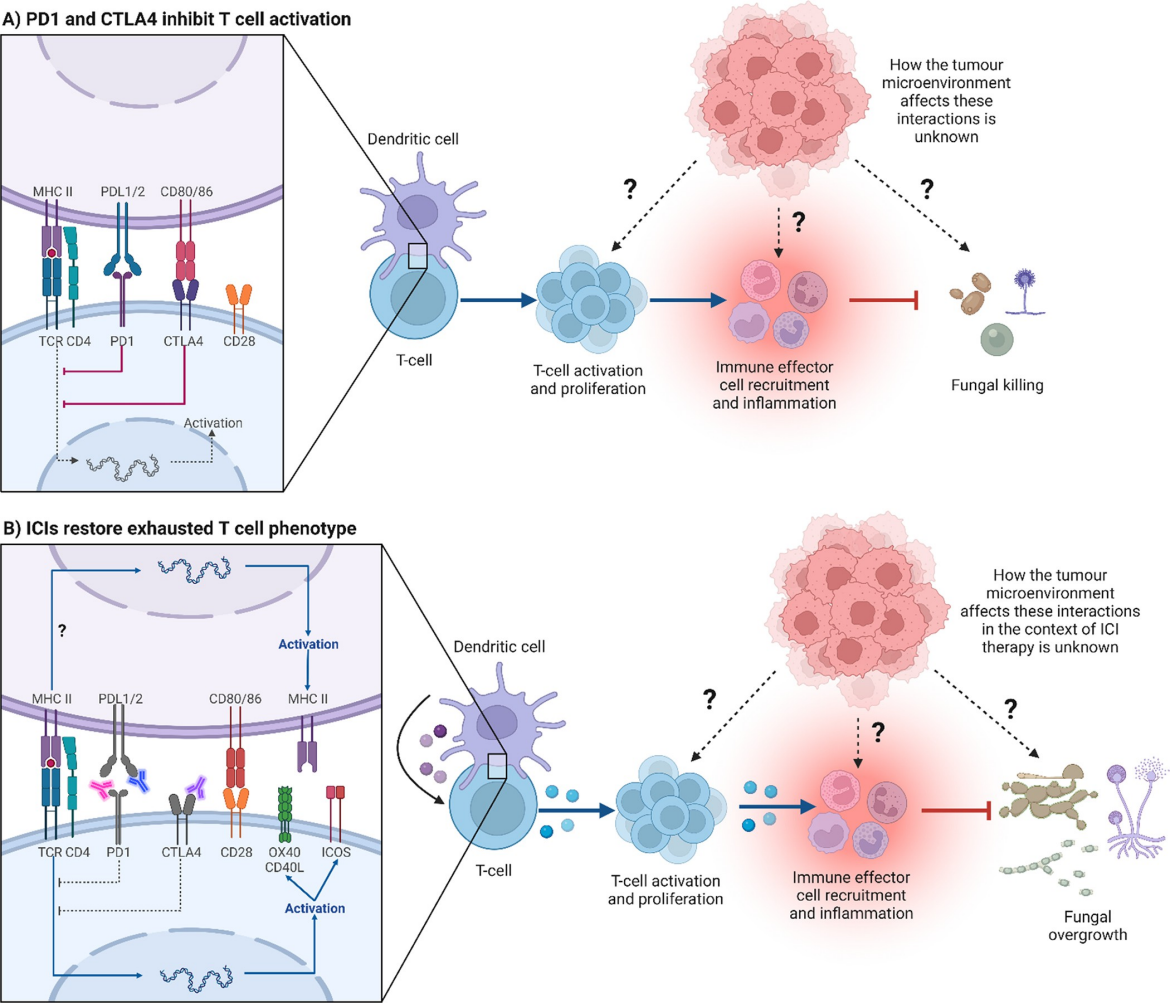
ICIs effect on T-cell function and antifungal immunity. (A) Inhibitory receptors such as PD1 and CTLA4 inhibit T-cell activation and prevent excessive immune activation during TCR and CD28 engagement. (B) ICIs (anti-CTLA-4 and anti-PD1/PD-L1/PD-L2 antibodies) can overcome these negative regulatory mechanisms by blocking the interaction between inhibitory receptors and their ligands. In the context of fungal infections (including primary fungal sepsis induced by systemic infection with *C*. *albicans*, secondary fungal sepsis occurring after sublethal cecal ligation and puncture, acute infection with *Histoplasma capsulatum*, persistent cryptococcal lung infection, invasive pulmonary mucormycosis, invasive pulmonary aspergillosis, and *Pneumocystis* pneumonia), ICI therapy has been associated with fungal clearance. Importantly, this has not yet been shown in humans. However, this protective antifungal response observed in the absence of tumour (being associated with increased expression of MHC-II on APCs, increased expression of co-signalling molecules and IFN-γ production by T cells, and increased levels of proinflammatory cytokines and chemokines) could shift towards a non-protective inflammatory response when an active tumour is present. Image was created with Biorender.com. APC, antigen presenting cell; ICI, immune checkpoint inhibitor; TCR, T cell receptor.

T-cell exhaustion is a tolerance mechanism that occurs in settings of persistent antigen exposure such as in chronic infections or in certain cancers. Compared to fully activated functional effector T cells, exhausted cells are desensitised to antigen stimulation, instead display defective responses including a high expression level of inhibitory receptors, decreased cytokine expression, altered epigenetic and metabolic transcriptional states, and inability to enter a quiescence-like state seen in memory T cells [1]. Inhibitory receptors serve as a checkpoint that prevents excessive immune activation during T cell receptor (TCR) and CD28 engagement (Fig 1). Indeed, in the clinic, some cancer patients treated with antibodies targeting inhibitory receptors commonly known as immune checkpoint inhibitors (ICIs) including programmed cell protein death 1 (PD-1) [anti-PD-1 (pembrolizumab, nivolumab, and cemiplimab)], cytotoxic T lymphocyte antigen-4 (CTLA-4) [anti-CTLA-4 antibody (ipilimumab)], LAG3 and others (reviewed elsewhere [2]), showed improved survival associated with restored effector T-cell function and improved anticancer immunity. Following T-cell activation, CTLA-4, which has a higher affinity to B7 molecules (CD80 and CD86, expressed on antigen presenting cells (APCs)) is translocated to the cell membrane and outcompetes CD28, effectively inhibiting further T-cell activation and proliferation (Fig 1). In addition, CTLA-4 is critical for regulatory T cell (Treg) function, which also acts to dampen immune response. PD-1, following interaction with its ligands PD-L1 or PD-L2, imposes negative regulation via recruitment of the tyrosine phosphatase SHP2 that attenuates TCR and CD28 signalling [3]. These negative regulatory mechanisms are overcome by anti-CTLA-4 and anti-PD1/PD-L1/PD-L2 blockade and other ICIs (Fig 1).

## 2. Do ICIs increase the risk of developing fungal diseases?

Since its introduction into clinical practice, numerous clinical case reports and case series show an increased frequency of invasive fungal diseases (IFDs) in patients with solid tumours, metastatic cancers, and lymphoma treated with ICIs (S1 References). This is remarkable, as those patient groups are historically characterised by a very low risk of IFDs compared to patients with leukaemias and haematopoietic stem cell transplantation [4]. Until now, 61 patients are reported in the literature who developed an IFD associated with the use of ICIs (S1 References). The majority of those infections were localised in the lung and caused by *Pneumocystic jirovecii* (*n* = 15) or *Aspergillus fumigatus* (*n* = 15). Twenty-nine IFDs were reported to be caused by *Candida albicans*, including 13 where isolation of *C*. *albicans* was associated with respiratory disease. Isolation of *C*. *albicans* from respiratory samples is not of clinical significance, unless there is histological evidence of tissue invasion and clinical signs and symptoms. As these details could not be retrieved from the literature, it is highly likely that these patients didn’t suffer from an IFD. One patient developed blastomycosis and 1 fusariosis. In the absence of denominator data, these case reports will not provide an answer to the question if ICI therapy increase the risk of IFDs. Furthermore, consideration needs also to be given to the fact that the association might be either a direct or indirect effect.

Immune-related adverse events (irAEs) are a common side effect of ICIs and are treated with corticosteroids and other immunosuppressive agents, which are well-known risk factors for the development of IFDs. Out of the 61 patients, 22 patients were treated for irAEs when developing an IFD. Of note, in a cohort study of 327 patients receiving ICIs, 30 fungal infections (15% of total infections) were observed with the only significant risk factor being the use of ICIs, while the use of corticosteroids was not a risk factor [5]. Presentation or exacerbation of chronic manifestations of fungal disease (chronic pulmonary aspergillosis, allergic bronchopulmonary aspergillosis, and fungal sinusitis) was observed in 4 patients when treatment with ICIs was initiated [6–9]. These clinical observations do point to diverse but related mechanisms rendering the patient susceptible to develop IFDs: directly immunotherapy driven, irAE treatment driven (immunosuppression), and/or dysregulated immunity (hyper inflammatory responses).

## 3. How might ICIs affect the risk to fungal diseases?

Immunity against fungi relies on C-type lectin receptors (CLRs) and the downstream adaptor CARD9. Defects in CLR-CARD9 signalling axis is associated with reduced phagocytosis and killing of fungal cells, altered inflammatory cytokine and chemokine expression, and impaired T cell- and antibody-mediated immunity [10,11]. Several studies have reported a T-cell exhaustion phenotype during models of fungal infection (including primary fungal sepsis induced by systemic infection with *C*. *albicans*, secondary fungal sepsis occurring after sublethal cecal ligation and puncture, acute infection with *Histoplasma capsulatum*, persistent cryptococcal lung infection, invasive pulmonary mucormycosis, invasive pulmonary aspergillosis, and *Pneumocystis* pneumonia), and thus a reasonable expectation that ICI therapy could improve fungal infection outcome [12–19]. In line with this, administration of either anti-PD1, anti-PD-L1, or anti-CTLA-4 antibodies during experimental models of fungal infections (as mentioned above) was associated with fungal clearance and improved survival outcome. Mechanistically, during systemic *Candida* spp. infection, the protective effect of anti-PD1 therapy was associated with increased expression of MHC-II on APCs and increased IFN-γ producing T cells [12]. Likewise, PD1 deficiency enhanced macrophage activation and promoted pulmonary Th1/Th17 responses during *Pneumocystis* pneumonia [19]. However, in a model of persistent cryptococcal lung infection, anti-PD1 treatment did not alter myeloid cell activation and rather reduced expression of IL-5 and IL-10 by lung leukocytes and up-regulated expression of the co-signalling molecule OX40 by Th1/Th17 cells [15]. Whereas, in a model of invasive pulmonary aspergillosis, the increased levels of proinflammatory cytokines and chemokines resulted in augmented recruitment of neutrophils to the lung [18]. Importantly, the type of T helper cell response varies with the model. In a model of invasive pulmonary mucormycosis, anti-PD1 therapy elicited pulmonary release of proinflammatory chemokines and cytokines including cytokines associated with type 2 (i.e., IL-4 and IL-13) and regulatory T-cell responses (i.e., IL-10) [17] (Fig 1) [12–19]. While the use of ICIs in experimental fungal infection models shows a favourable outcome associated with enhanced antifungal immunity, observations of the occurrence of IFDs in cancer patients receiving ICIs points to negative impact on the antifungal immune response resulting in the inability to combat the fungal infection. A better understanding of the antifungal effector immune cell phenotypes and the expression profiles of their inhibitory receptors during different cancer cell types is required (Fig 1). Therefore, novel models to study the role of ICIs on IFDs in the settings of cancer therapy need to be established.

Extensive studies have been done to understand, predict, and stratify different cancer patients’ responsiveness to ICI therapy. However, the literature is very limited when it comes to predicting susceptibility to subsequent fungal infections. No studies so far have addressed the effect of ICIs on the course of fungal infections. Therefore, more preclinical and clinical studies are required to address the impact of ICI in antifungal immunity in the context of cancer immunotherapy and chemotherapy.

## 4. Could ICI be a possible adjunctive therapy to combat fungal diseases?

Interestingly, there are a couple of case reports of patients with mucormycosis that were successfully treated with a combination therapy of anti-PD1 and IFN-γ [20,21]. The first was a patient that sustained pelvic and abdominal damage, pulmonary contusion, and second-degree burns, who developed extensive abdominal mucormycosis unresponsive to conventional therapy with amphotericin B and posaconazole. Treatment with IFN-γ and nivolumab led to increased absolute lymphocyte counts, CD8+ T cells and monocyte HLA-DR expression while decreased T cell PD-1 expression [20]. The second was an immunosuppressed haematological patient suffering from mucormycosis and aspergillosis coinfection unresponsive to antifungal therapy who was subsequently treated with nivolumab and IFN-γ therapy. Treatment significantly improved patient’s condition, which was associated with a decreased expression of PD-1 and CTLA-4 and an increased expression of Tim3, OX40, and CD40L [21]. Ex vivo, human PBMCs from patients with paracoccidioidomycosis have also been shown to exhibit restored T-cell proliferation and IFN-γ production following CTLA-4 and Fas-FasL blockage [22]. However, whether those changes in co-signalling molecules are directly responsible for controlling the fungal pathogen is unknown and will require further investigation.

## 5. What human CTLA-4 and PD-1 deficiencies could tell us about ICIs and the risk to fungal diseases?

Autosomal dominant CTLA-4 haploinsufficiency, described for the first time in 2014, results in an autosomal dominant immune dysregulation syndrome [23]. Lipopolysaccharide-responsive and beige-like anchor protein (LRBA) plays a crucial role in CTLA-4 regulation and LRBA deficiency is associated with deficient CTLA-4 function [24]. Clinical phenotypes of these 2 inborn errors share many commonalities, with autoimmunity being the hallmark of these diseases. Severe infections are reported in 30% and 44% of patients with CTLA-4 and LRBA deficiency, respectively, with approx. 10% of infections caused by *Candida* or *Aspergillus* species [25]. Only 1 patient has been described with an inherited PD-1 deficiency who suffered from infancy onwards from autoimmunity, abdominal tuberculosis and died at the age of 11 years due to autoimmune pneumonitis [26]. These so-called “experiments of nature” might provide us with a deeper understanding of the potential consequences of inhibiting specific immune cell checkpoints to optimise cancer treatment and prevent fungal infections.

## 6. Could the mycobiome influence the risk to fungal diseases in the context of ICIs?

As more immune checkpoint molecules are being identified as targets for augmenting T cell functionality for cancer therapy [27], careful consideration needs to be made on their impact on homeostatic immune mechanisms that control fungal commensals, still being uncovered [28,29]. Of particular concern is the off-target impact of removing immune checkpoints at mucosal tissue sites, which serve as a reservoir for commensal fungi like *Candida* in the skin and gut, and the lungs that are exposed to large volumes of antigens including *Aspergillus* spores. Immune tolerance at these sites is most at play, thus these perturbations need further investigation. The observation that diverse fungal DNA can be found in various tumours [30,31] need to be taken into account in this complex interplay.

In summary, we have highlighted the clinical observations suggesting an association between the occurrence of IFDs and ICI therapy. If ICI therapy increases the susceptibility to develop IFD directly or indirectly due to use of immunosuppressive treatment for irAEs or a combination of both is not clear. The development of IFDs and/or exacerbation of preexisting chronic fungal infections should be considered if a patient on ICI therapy presents with signs of infection and/or inflammation. Experimental models will need to be developed to assess how ICI affect antifungal immunity in the host with specific cancer-associated IFDs. We suggest a way forward to address these needs to include [1] establishing murine cancer models with relevant fungal microbes that expand in these cancers and investigate fungal disease and the effect of currently used ICIs, and [2] exploiting high-dimensional phenotyping and determination of antifungal immune responses in these contexts, and [3] assessing spatial and temporal resolution of exhaustion maker profiles in the complex tumour microenvironments compared to peri-tumoural sites of antifungal T cells versus antitumour T cells. No doubt, future studies will help to optimise these immune therapies while minimising the undesired side effects.

## Supporting information

S1 References List of clinical studies reporting the development of IFD associated with the use of ICIs. (DOCX)
