# Supplementary material for: Unmasking a fungal fire
Source: PLoS Pathog. 2023 May 18;19(5):e1011355. doi: 10.1371/journal.ppat.1011355 (PMC10194863; doi:10.1371/journal.ppat.1011355)
Supplement: S1 References — (DOCX) [file ppat.1011355.s001.docx]

**Supporting information**

**S1 References**

1. Arriola E, Wheater M, Krishnan R, Smart J, Foria V, Ottensmeier C. Immunosuppression for ipilimumab-related toxicity can cause pneumocystis pneumonia but spare antitumor immune control. Oncoimmunology. 2015 Oct;4(10):e1040218.

2. Del Castillo M, Romero FA, Arguello E, Kyi C, Postow MA, Redelman-Sidi G. The Spectrum of Serious Infections Among Patients Receiving Immune Checkpoint Blockade for the Treatment of Melanoma. Clin Infect Dis. 2016 Dec 1;63(11):1490-3.

3. Do MH, Barrios DM, Phillips GS, Postow MA, Warner AB, Rosenberg JE, et al. Dermatologic infections in cancer patients treated with checkpoint inhibitors. J Am Acad Dermatol. 2021 Dec;85(6):1528-36.

4. Ferguson I, Heberton M, Compton L, Keller J, Cornelius L. Disseminated blastomycosis in a patient on pembrolizumab for metastatic melanoma. JAAD Case Rep. 2019 Jul;5(7):580-1.

5. Fujita K, Kim YH, Kanai O, Yoshida H, Mio T, Hirai T. Emerging concerns of infectious diseases in lung cancer patients receiving immune checkpoint inhibitor therapy. Respir Med. 2019 Jan;146:66-70.

6. Gupta A, Tun A, Ticona K, Baqui A, Guevara E. Invasive Aspergillosis in a Patient with Stage III (or 3a or 3b) Non-Small-Cell Lung Cancer Treated with Durvalumab. Case Rep Oncol Med. 2019;2019:2178925.

7. Inthasot V, Bruyneel M, Muylle I, Ninane V. Severe pulmonary infections complicating nivolumab treatment for lung cancer: a report of two cases. Acta Clin Belg. 2020 Aug;75(4):308-10.

8. Karam JD, Noel N, Voisin AL, Lanoy E, Michot JM, Lambotte O. Infectious complications in patients treated with immune checkpoint inhibitors. Eur J Cancer. 2020 Dec;141:137-42.

9. Krane NA, Beswick DM, Sauer D, Detwiller K, Shindo M. Allergic Fungal Sinusitis Imitating an Aggressive Skull Base Lesion in the Setting of Pembrolizumab Immunotherapy. Ann Otol Rhinol Laryngol. 2021 Jan;130(1):108-11.

10. Kyi C, Hellmann MD, Wolchok JD, Chapman PB, Postow MA. Opportunistic infections in patients treated with immunotherapy for cancer. J Immunother Cancer. 2014;2:19.

11. Liu Z, Liu T, Zhang X, Si X, Wang H, Zhang J, et al. Opportunistic infections complicating immunotherapy for non-small cell lung cancer. Thorac Cancer. 2020 Jun;11(6):1689-94.

12. Lord JD, Hackman RC, Moklebust A, Thompson JA, Higano CS, Chielens D, et al. Refractory colitis following anti-CTLA4 antibody therapy: analysis of mucosal FOXP3+ T cells. Dig Dis Sci. 2010 May;55(5):1396-405.

13. Malek AE, Taremi M, Spallone A, Alvarez-Cardona JJ, Kontoyiannis DP. Necrotizing soft tissue invasive aspergillosis in a cancer patient treated with immunosupressants due to checkpoint inhibitor-induced hepatitis. J Infect. 2020 Feb;80(2):232-54.

14. Oltolini C, Ripa M, Andolina A, Brioschi E, Cilla M, Petrella G, et al. Invasive Pulmonary Aspergillosis Complicated by Carbapenem-Resistant Pseudomonas aeruginosa Infection During Pembrolizumab Immunotherapy for Metastatic Lung Adenocarcinoma: Case Report and Review of the Literature. Mycopathologia. 2019 Feb;184(1):181-5.

15. Pradere P, Michot JM, Champiat S, Danlos FX, Marabelle A, Lambotte O, et al. Allergic broncho-pulmonary aspergillosis following treatment with an anti-programmed cell death protein 1 monoclonal antibody therapy. Eur J Cancer. 2017 Apr;75:308-9.

16. Sadek M, Loizidou A, Drowart A, Van den Wijngaert S, Gomez-Galdon M, Aspeslagh S. Pneumocystis Infection in Two Patients Treated with Both Immune Checkpoint Inhibitor and Corticoids. J Immunother Precis Oncol. 2020 Feb;3(1):27-30.

17. Schwarz M, Kocher F, Niedersuess-Beke D, Rudzki J, Hochmair M, Widmann G, et al. Immunosuppression for Immune Checkpoint-related Toxicity Can Cause Pneumocystis Jirovecii Pneumonia (PJP) in Non-small-cell Lung Cancer (NSCLC): A Report of 2 Cases. Clin Lung Cancer. 2019 May;20(3):e247-e50.

18. Taima K, Tanaka H, Itoga M, Ishioka Y, Kurose A, Tasaka S. Destroyed lung due to sustained inflammation after chemoradiotherapy followed by durvalumab. Respirol Case Rep. 2020 Jul;8(5):e00580.

19. Tokumo K, Masuda T, Miyama T, Miura S, Yamaguchi K, Sakamoto S, et al. Nivolumab-induced severe pancytopenia in a patient with lung adenocarcinoma. Lung Cancer. 2018 May;119:21-4.

20. Uchida N, Fujita K, Nakatani K, Mio T. Acute progression of aspergillosis in a patient with lung cancer receiving nivolumab. Respirol Case Rep. 2018 Feb;6(2):e00289.
